# T cell-inspired therapeutic delivery platforms: From nanomedicines to cell therapy

**DOI:** 10.1016/j.mtbio.2026.102909

**Published:** 2026-02-10

**Authors:** Nasrullah Jan, Hassan Shah, Safiullah Khan, Naveed Ullah Khan, Yan Li, Xinwei Zhang, Guixiu Shi

**Affiliations:** a Department of Rheumatology and Clinical Immunology, The First Affiliated Hospital of Xiamen University, School of Medicine, Xiamen University, Xiamen, PR China; b Xiamen Municipal Clinical Research Center for Immune Diseases, Xiamen, PR China; c Xiamen Key Laboratory of Rheumatology and Clinical Immunology, Xiamen, PR China; d Department of Minimally Invasive Interventional Radiology, The Second Affiliated Hospital, School of Biomedical Engineering, Guangzhou Medical University, Guangzhou, Guangdong, PR China; e Department of Pharmacy, Iqra University, H-9 Campus Islamabad, Islamabad, Pakistan; f Affiliated Hospital Guangdong Medical University, Zhanjiang, PR China

**Keywords:** T cell, Drug delivery, Nanomedicines, CAR T cell therapy

## Abstract

Targeted nanodrug delivery has garnered significant interest as a carrier for drugs, genes, and vaccines. Despite their clinical potential, these nanocarriers face substantial challenges due to their exogenous nature. These challenges can be addressed by employing T cell-inspired approaches for targeted therapies. T cell-inspired approaches—including T cell membrane-coated nanoparticles, T cell-derived exosomes, T cell hitchhiking, and chimeric antigen receptor (CAR)-T cells—exhibit remarkable properties such as inherent biocompatibility and biodegradability, prolonged circulation lifespan, and the ability to traverse biological barriers. Utilizing T cells as delivery vehicles enables prolonged circulation time and targeted drug transport, along with reduced toxicity to cells and tissues. This review explores innovative T cell-derived approaches, including T cell membrane-coated nanoparticles, T cell-derived exosomes, T cell hitchhiking, and CAR-T cells. We discuss how these methods improve biodistribution, tissue penetration, and immune evasion while preserving T cell functionality in cancer therapies, autoimmune disorders, cardiovascular diseases, and infectious diseases. By comparing conventional nanomedicine approaches with emerging T cell-based delivery systems, this review explores the transformative capability of T cell-inspired delivery in enhancing therapeutic outcomes. Finally, we address current limitations and future directions, including advanced engineering techniques, which could further refine this promising approaches.

## Highlights

• Cell-based nanodelivery systems offer inherent biocompatibility, prolonged circulation, and targeted delivery.
• T cell-inspired approaches, including membrane coating, exosomes, hitchhiking, and CAR-T cells are reviewed.
• Applications of T cell-inspired approaches for various diseases are discussed, along with current limitations and future opportunities.

## Graphical abstract

**Figure:**
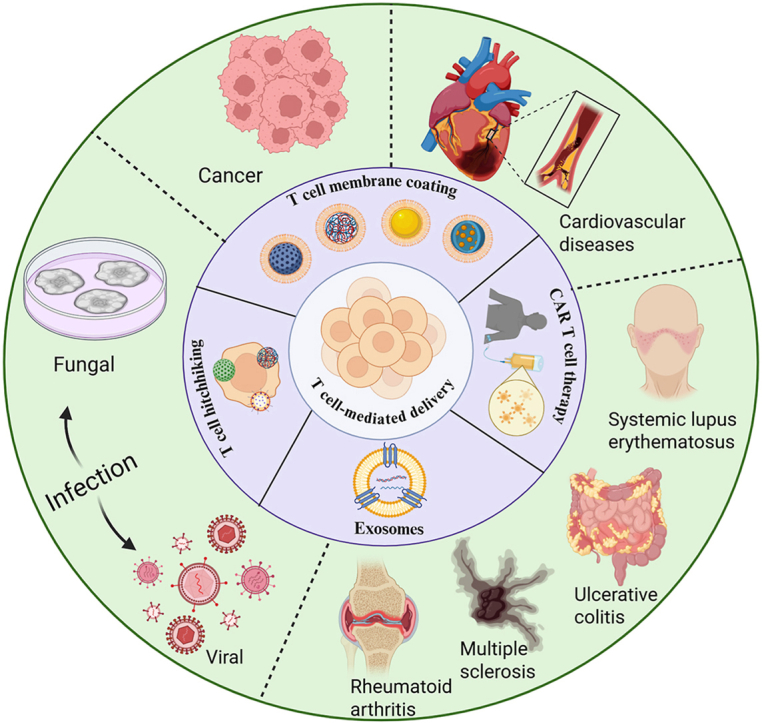

## 1 Introduction

Nanoparticulate carrier systems have gathered significant interest as a carriers for drugs, genes, or vaccines due to their favorable biocompatibility, low toxicity, and the ability to achieve controlled release in vivo [1]. Nonetheless, their practical applications remain constrained, as nanoformulations, similar to other foreign materials, are identified by the immune system as exogenous entities and are rapidly cleared by mononuclear phagocyte system (MPS) from circulation [2]. The predominant strategy to mitigate MPS-mediated clearance involves the modification of nanoparticle surfaces with polyethylene glycol (PEG) [3]. However, PEGylated nanocarriers may also trigger the accelerated blood clearance (ABC) phenomenon, that significantly compromise their safety and efficacy [4]. Consequently, the rapid systemic clearance of nanocarriers and their limited capacity to navigate various physiological barriers must be addressed for clinical translations of these nanocarriers.

Cell-mediated drug delivery systems, which utilize circulating cells such as erythrocytes, immune cells, and stem cells, have been employed as a vehicles for therapeutic agents [5]. These circulating cells exhibit remarkable properties, including inherent biocompatibility and biodegradability, an extended circulation lifespan, and the ability to traverse biological barriers. Furthermore, these cells possess natural homing capabilities that allow them to travel and concentrate at inflammation, infection, and tumors sites, making them an ideal candidates for the development of targeted drug delivery systems [6]. Among the various cell types, T lymphocytes are the main players of cell-mediated immunity and can be classified into *CD8*^*+*^ T cells (cytotoxic), *CD4*^*+*^ T cells (helper), memory T cells, and regulatory T cells (Tregs) according to their functions [7]. T cells play a vital role in immunotherapy due to their three primary functions: the direct destruction of foreign pathogens, the activation of adjacent immune cells through cytokine release, and the enhancement of B cell responses [8]. A prevalent method involves isolating T cells from a patient own blood and conjugating CARs to their surface. This modification enables T cells to effectively target tumor sites, as the expression of specific antigens on the T cells guides their localization to the tumor [9]. However, a significant challenge in ex vivo cell-based therapies is the rapid decline in the viability and functionality of the genetically modified T cells following transplantation [10]. To circumvent the intrinsic viability limitations of transplanted cells, an innovative in vivo strategy was proposed. This approach involves the systemic administration of nanoparticles designed to bind specifically to circulating T cells. Rather than aiming to improve the long-term survival of the transplanted cells themselves, this method leverages the transient presence of T cells as delivery vehicles to ferry therapeutic cargo to target sites [11]. Beyond the strategy of attaching nanoparticles to the cell surface, another potential approach involves loading nanoparticles within T cells [12]. Similarly, T-cell membrane-coated nanoparticles represent advanced drug delivery systems that confer several advantages to the nanoparticles, including extended circulation time, an expanded range of drug targets, controlled release mechanisms, targeted cellular interactions, and reduced in vivo toxicity [13]. These approaches suggest that T lymphocytes may serve as a promising strategy for the targeted carrying of therapeutic agents.

Herein, we provide a comprehensive summary of emerging strategies that harness T cells for targeted drug delivery. These innovative approaches includes T cell membrane-coated nanoparticles, T cell hitchhiking, T cell-derived exosomes, and CAR-T cell therapy. This comprehensive overview highlights the versatile role of T cells as both delivery vehicles and therapeutic agents, emphasizing their potential to revolutionize treatment modalities in diverse clinical settings.

## 2 T cell biology and functions: Origins, maturation, and immunological roles

T cells are key player of adaptive immune response, characterized by unparalleled specificity, long-term memory, and precise regulatory functions. These characteristics are essential for safeguarding the body against invading pathogens and abnormal cells, as well as maintaining immune homeostasis [14]. Unlike other immune cells such as neutrophils and macrophages, which rely on preprogrammed pattern recognition receptors to identify common microbial structures [15], T cells mount highly specific responses by recognizing and responding only to particular antigens presented by other cells. This specificity enables the targeted elimination of threats and the development of long-lasting immunity that defends against recurrent infections [16].

### 2.1 The origin of T cells: From hematopoietic stem cells to lymphoid progenitors

**Fig. 1:**
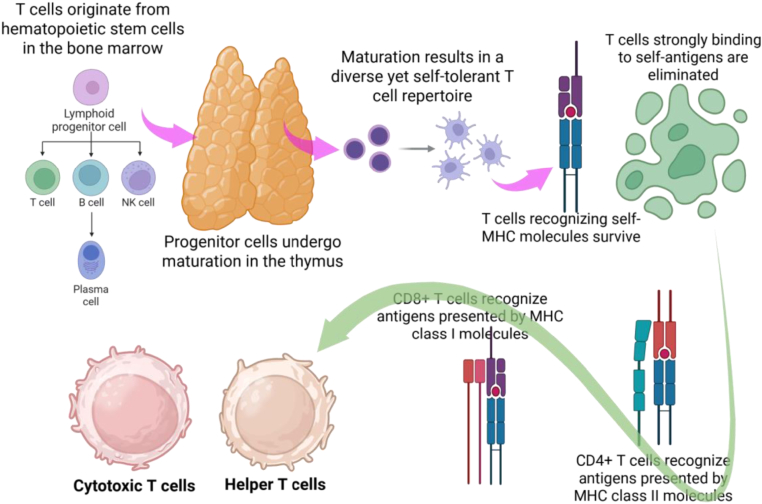
Origins, maturation, and immunological roles of T cells. T lymphocytes originate from bone marrow and matured in thymus. T cells recognize self-MHC molecules survive and converted into CD4^+^ T cells or CD8^+^ T cells based on recognition of MHC class I and II molecules. (www.BioRender.com).

The life cycle of T lymphocytes begins in the bone marrow (Fig. 1), the primary site of hematopoiesis in adult mammals. Here, multipotent hematopoietic stem cells (HSCs) serve as the origin. These stem cells have two defining characteristics: self-renewal, which allows them to maintain a stable population throughout life, and pluripotency, which enables them to discriminate into all types of blood cells, including both lymphoid and myeloid lineages [17]. For T cell development, HSCs first differentiate into lymphoid progenitor cells, representing a crucial branching point in lymphoid lineage commitment. At this stage, lymphoid progenitors are not yet functionally mature and retain the potential to differentiate into the three major cell types of the lymphoid lineage: NK cells, T cells, and B cells [18]. The fate of these progenitors is determined by a combination of intrinsic genetic programs and extrinsic signals from the bone marrow microenvironment [19]. Notably, while B cells and NK cells mature within the bone marrow, T cell progenitors follow a distinct path: they exit the bone marrow via systemic circulation and migrate to the thymus. This migration marks the initiation of T cell-specific maturation, a process critical for shaping their ability to recognize threats while avoiding attacks on healthy self-tissues [20].

### 2.2 T cell maturation in the thymus: Selection for self-tolerance and MHC recognition

The thymus serves as a "training ground" for T cell progenitors, where only functional, self-tolerant T cells are released into the peripheral immune system by two-step selection process. This maturation process is tightly regulated by thymic epithelial cells, DCs, and other stromal cells within the thymus, which provide the necessary signals and antigens for selection [21]. The first critical step in thymic maturation is positive selection, which ensures that T cells can recognize MHC molecules—cell surface proteins that present antigens to T cells. During positive selection, T cell progenitors interact with MHC molecules (either MHC class I or class II) of thymic epithelial cells [20]. T cells that weakly bind to these self-MHC molecules receive survival signals. This weak binding is essential because it guarantees that the T cell will be able to interact with MHC molecules on other cells (such as antigen-presenting cells or infected somatic cells) later in the periphery. In contrast, T lymphocytes that fail to bind self-MHC molecules do not obtain survival signals and undergo programmed cell death (apoptosis). This step effectively eliminates T cells that cannot recognize MHC-antigen complexes, ensuring that only "functional" T cells proceed to the next stage [22]. The second, equally crucial step is negative selection, which prevents autoimmunity by eliminating self-reactive T cells [23]. Following positive selection, the surviving T cells interact with DCs and thymic epithelial cells that present a diverse array of self-antigens—proteins derived from healthy body tissues—bound to MHC molecules. T cells that bind strongly to these self-antigen-MHC complexes are considered potentially harmful, as they may attack healthy self-cells and trigger autoimmune diseases. These strongly self-reactive T cells are actively eliminated through apoptosis [24]. Together, positive and negative selection generate a diversified yet self-tolerant T cell repertoire. By the end of thymic maturation, T lymphocytes are classified into two major subsets based on the co-receptors they express: CD4^+^ T cells and CD8^+^ T cells. This subset division is closely linked to their ability to recognize different MHC molecules: CD4^+^ T cells specifically recognize MHC class II presented antigens, whereas CD8^+^ T cells recognize antigens presented by MHC class I molecules. This specialization forms the basis for their discrete functional roles in the peripheral immune system [25].

### 2.3 Functional specialization of mature T cells: Orchestrating immune responses

Upon maturation, T cells exit the thymus and enter into peripheral lymphoid organs, such as spleen and lymph nodes. Where, they assume specialized roles that are critical for adaptive immune defense. CD8^+^ T cells, commonly known as CTLs (cytotoxic T lymphocytes), are the primary effectors responsible for targeted cell killing. Their hallmark is the ability to identify antigens presented by MHC class I molecules—a type of MHC molecule expressed on nearly all nucleated somatic cells [26]. When a somatic cell are infected with virus or undergoes malignant transformation, it degrades viral or abnormal proteins into small peptides. These peptides are displayed on the cell surface due to loading onto MHC class I. CD8^+^ T cells that recognize the complex of these antigen-MHC class I become activated, proliferate rapidly, and differentiate into effector CTLs [27]. These effector cells subsequently release cytotoxic molecules—such as perforins and granzymes—that puncture the membrane of the infected or cancerous cell, inducing apoptosis and eliminating the threat. This mechanism is especially critical for controlling viral infections (e.g., influenza or herpes) and suppressing tumor growth [28]. CD4^+^ T cells primarily function as "orchestrators" of the immune response and are commonly known as helper T cells (Th cells). They recognize the complex of antigens-MHC class II, which are expressed on professional APCs, including DCs, macrophages, and B cells. Upon activation, CD4^+^ T cells transform into various subsets (e.g., Th1, Th2, Th17, and Tregs) that secrete distinct cytokines, acting as signaling molecules to coordinate other immune cells [29]. For example, Th1 cells secrete cytokines that activate macrophages to eliminate intracellular pathogens [30], while Th2 cells promote B cell activation and transformation into plasma cells—cells that produce antibodies to neutralize extracellular pathogens like bacteria and viruses [31]. Additionally, Tregs, a subset of CD4^+^ T cells, play a key role in preserving immune tolerence and preventing autoimmunity, thereby ensuring that the immune system does not overreact to harmless antigens or attack healthy tissues [32].

## 3 T cell-inspired therapeutic delivery

### 3.1 T cell hitchhiking

**Fig. 2:**
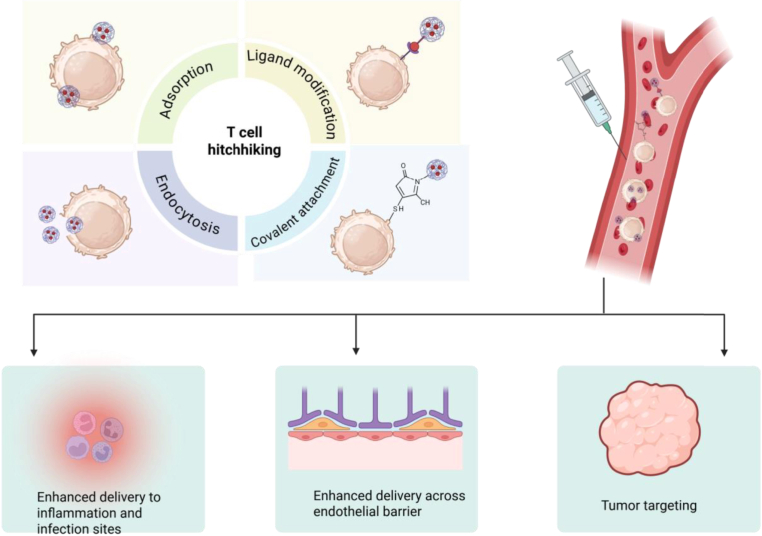
Schematic illustration of targeted delivery of T-cell hitchhiked nanoparticle. Nanoparticles hitchhiked to T cell involves adsorption, endocytosis, covalent attachment, and ligand modification. Adsorption of nanoparticle to cell surface is simple technique that can be obtained by hydrogen bonding, electrostatic force, hydrophobic interaction or Van der Waals force between nanoparticles and cell. Endocytosis is the exploiting to phagocytic function of cell to engulf the nanoparticles. Covalent attachment is the covalent bonding between groups present on the cargo and cell membrane. Ligand attachment utilizes naturally occurring ligands on cell surface. The hitchhiked nanoparticles to T cell has increased tumor targeting capability, can easily cross the endothelial barrier, and enhanced delivery to inflammation and infection sites. (www.BioRender.com).

Cellular hitchhiking represents the co-transportation of a secondary cargo alongside a primary cargo that is already in motion. During this process, the secondary payload is effectively recognized as a component of the cell while it circulates, thereby evading clearance mechanisms [33]. The criteria that define cellular hitchhiking include the interactions between the primary and secondary cargo, their simultaneous migration, and the transportation driven by the movement of the primary cargo rather than the secondary [34]. By masquerading the secondary cargo as 'self,' cellular hitchhiking facilitates evasion of clearance and traversal of endothelial barriers, thereby prolonging circulation time and minimizing toxicity through the reduction of off-target interactions. Consequently, the phenomenon of cellular hitchhiking in the context of nanoparticles—where synthetic nanoparticles associate with circulating cells for targeted delivery—holds significant potential for enhancing the therapeutic efficacy of nanoparticles-based treatments [35]. T cell hitchhiking has shown promise not only in enhancing the cytotoxic capabilities of T cells but also in improving their proliferation and persistence at tumor sites [36]. T cells possess specialized receptors that help them to recognize and respond to tumor antigens. The phenomenon of cellular hitchhiking can increase the perseverence and anti-tumor efficacy of T cells at tumor sites [37]. Nanoparticles hitchhiked to T cell involves adsorption, endocytosis, covalent attachment, and ligand modification (Fig. 2). Adsorption of nanoparticle to cell surface is simple technique that can be obtained by hydrogen bonding, electrostatic force, hydrophobic interaction or Van der Waals force between nanoparticles and cell. Endocytosis is the exploiting to phagocytic function of cell to engulf the nanoparticles. Covalent attachment is the covalent bonding between groups present on the cargo and cell membrane. Ligand attachment utilizes naturally occurring ligands on cell surface [34]. In the context of T lymphocytes, a PD-1 antibody-modified metal-organic framework combined with liposomal nanoparticles interacts with PD-1 antigens present on T cells during their circulation, effectively tethering the nanoparticles to the T cells. Once the T cells are transported into tumor regions, the nanoparticles facilitate the release of haemoglobin and catalase, which alleviates tumor hypoxia and subsequently activates cytotoxic T cells, thereby contributing to the treatment of colorectal cancer [38].

### 3.2 T cell membrane-coated nanoparticles

**Fig. 3:**
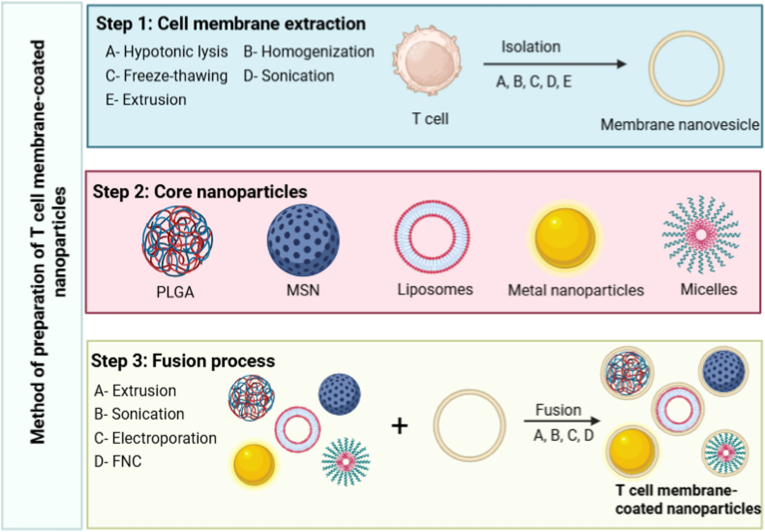
Schematic illustration of method of preparation of T cell membrane-coated nanoparticles. The method of preparation involves, **Step 1**: Extraction of membrane from T cell. **Step 2**: The core nanoparticles and **Step 3**: the coating of extracted cell membrane on core nanoparticles called fusion process. (www.BioRender.com.).

The technology of cell membrane coating employs surface modification of nanocarriers with the plasma membrane isolated from cell [39]. The intricate structure of a cell's membrane, which includes its proteins, lipids, and carbohydrates, can be effectively preserved by relocating the plasma membrane of the cell onto the surface of a nanoparticle. This process allows the resulting membrane-cloaked nanoparticle to exhibit various characteristics inherent to the source cell [40]. Cell membrane coating effectively addresses the limitations of synthetic nanoparticles, which are often recognized as foreign entities by biological systems. This approach confers inherent biocompatibility due to the naturally occurring outer layer, resulting in limited toxicity, immune escape, and enhanced stability. Consequently, these characteristics leads to prolonged circulation time in the bloodstream. This is attributed to the expression of membrane surface’ proteins that serve as self-recognition signals [41]. Additionally, one of the primary advantages of cell membrane coating is its ability to enhance targetability and specificity, stemming from the specific ligand-receptor interactions between cell membrane and target cells [42]. T-cell membrane-coated nanoparticles represent advanced drug delivery systems that enhance circulation time and mitigate rapid clearance by the immune system. Immune T-cells possess specific surface proteins that impart distinctive functionalities to biomimetic nanoparticles during the processes of membrane extraction and coating. These membrane proteins provide several advantages to the nanoparticles, including extended circulation duration, an expanded range of drug targets, controlled release mechanisms, targeted cellular interactions, and reduced in vivo toxicity [13,43]. The process of preparing T cell membrane-coated nanoparticles consists of three distinct stages (Fig. 3): the isolation of plasma membrane from the source cells, the fabrication of the core nanoparticles, and the subsequent coating of the extracted membrane on the core nanoparticles [40]. The disruption of cellular structures is a fundamental process for the extraction of T cell membrane vesicles. This process involves the creation of a lysate through the rupture of the cell membrane. Cell disruption techniques can be broadly categorized into two main types: chemical and physical methods. Chemical lysis employs various agents such as salts, buffers, detergents, and enzymes, and does not require mechanical scraping or crushing [44]. In contrast, physical methods include more rigorous approaches such as mechanical blending, compression, ultrasonic treatment, and the use of pestle and mortar. The most common techniques used for lysing T cells include hypotonic treatment, sonication, extrusion, and microfluidic electroporation. The core nanoparticles can be organic or inorganic [13]. The fusion of membrane nanovesicle with nanoparticles core is primarily achieved through three techniques: extrusion, sonication, and electroporation [45]. In the extrusion process, cell membrane vesicles and nanoparticles are sequentially co-extruded through membranes with varying pore sizes, resulting in the fusion of vesicles and particles. While this method is effective and yields stable results, it has limited suitability for large-scale production [46]. Sonication facilitates the fusion of co-incubated membrane vesicles with nanoparticles by using ultrasonic waves. However, it is essential to optimize the ultrasonic parameters to enhance fusion efficiency while minimizing protein denaturation and drug leakage [40]. Electroporation involves the creation of transient pores in the cell membrane by applying electrical pulses, allowing nanoparticles to enter. This technique offers significant advantages, including the preservation of cell membrane integrity and a reduction in the loss of membrane proteins [47]. Besides serving as novel drug carrier, T cell membrane-coated nanoparticles also enable multitude of therapeutic effects or enhance active targeting by incorporating specific ligands on their membrane. For targeted delivery, a multitude of techniques has been used to easily incorporate ligands into the cell membrane of T cells. These techniques include genetic engineering [48], chemical and physical modification [49], and membrane hybridization [50]. Moreover, T cell membrane coating presents a broad spectrum of therapeutic applications, facilitating the delivery of various treatments, including immunotherapy [51], gene therapy [52,53], chemotherapy [54], sonodynamic therapy [51], and photothermal therapy (PTT) [55].

### 3.3 T cell-derived exosomes

Exosomes are a type of extracellular vesicles characterized by their size, which ranges from 30 to 150 nm. These vesicles have the ability to penetrate cells, release their molecular cargo, and influence both physiological and pathological processes [56]. Exosomes can modulate gene expression in recipient cells by transferring specific mRNA, facilitate immune responses—either stimulating or suppressing them—aid in the excretion of substances from organs, and assist in the clearance of waste from the brain [57]. The capacity of exosomes to mediate various biological processes is harnessed for the targeted delivery of therapeutic cargos, aiming to mitigate systemic toxicity. Due to their natural origin, exosomes exhibit long-term accumulation in organs or tissues, resulting in negligible or absent systemic toxicity. Their compatibility with biological systems and organotropic properties make exosomes suitable as natural drug delivery vehicles, capable of transporting a diverse array of therapeutics, including genetic materials, into cells. Furthermore, their low immunogenicity and toxicity position exosomes as promising candidates for innovative drug carrier systems [58].

**Fig. 4:**
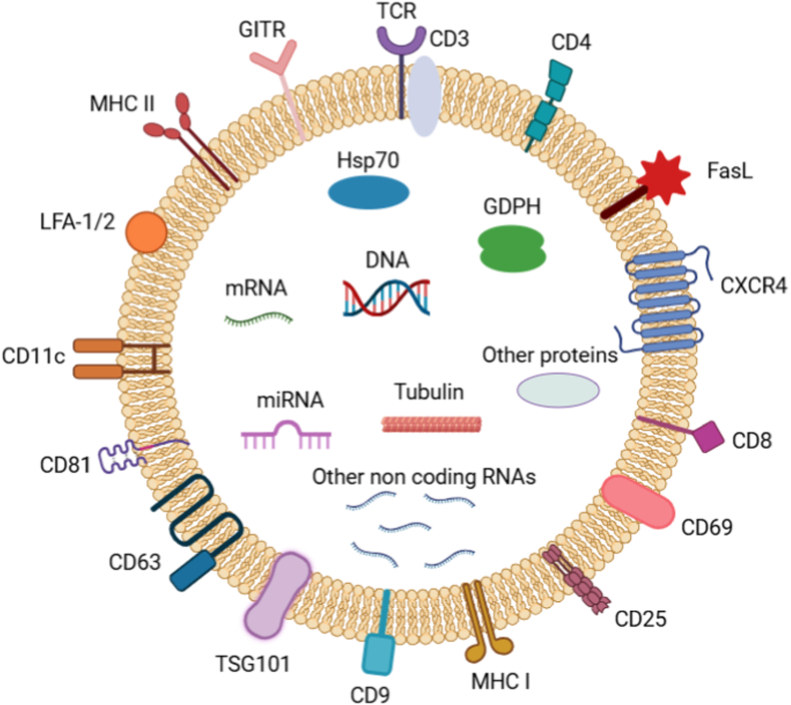
T cell-derived exosomes and their important surface biomarkers. Exosomes derived from T cells consist of surface and inter-molecules. The surface molecules includes TCR, CD3, CD4, CD8, CD9, CD11c, CD25, CD63, CD69, CD81, MHC-I, MHC-II, CXCR4, FasL, GITR, LFA1/2, and TSG101. The inter-molecules includes DNA, mRNA, miRNA, some non-coding RNA, GDPH, Hsp70, Tubulin and some other proteins. (www.BioRender.com.).

Naïve T lymphocytes are known to produce exosomes that play a vital role in modulating the activity of immune cells. These exosomes are distinguished by the expression of TCR (T cell receptors) and numerous adhesion molecules. Additionally, T cell-derived exosomes express a range of markers, including cluster of differentiation (CD)3, CD2, CD8, CD4, CD11c, CD69, CD25, lymphocyte function-associated antigen 1 (LFA-1), CXCR4, FAS ligand (FASL), and GITR [59] (Fig. 4). Exosomes generated from activated T cells in the presence of IL-2 have been shown to enhance the production of resting autologous cells, as they carry a substantial quantity of microRNAs [60]. The formation of immunological synapses is known to enhance the efficacy of exosome transfer between APCs and T cells. During this interaction, T cell exosomes facilitate the conversion of APCs into more effective one by transferring microRNAs [61].

Regulatory T lymphocytes (Tregs) are also significant producers of exosomes that express surface markers such as CD25, CTLA-4 and CD73, exerting immunosuppressive effects through various mechanisms [62]. The presence of CD73 on exosomes's suface is essential for immunomodulation, as it facilitates the production of adenosine, which is integral to the anti-inflammatory effect [61]. Exosomes derived from Treg cells inhibit the differentiation of Th1 cells and the synthesis of interferon-gamma (IFN-γ) by transferring microRNA to target cells. Additionally, many of the immunosuppressive effects of these exosomes are attributed to the presence of cytokines such as TGF-β and IL-10 [63]. Treg exosomes can also modulate DC function by delivering microRNAs that enhance IL-10 production while simultaneously reducing the production of TNF-α and IL-6 [64].

Considering the challenges inherent to CAR-T cell therapy, particularly cytokine release syndrome—which can manifest as nausea, headache, hypotension, and tachycardia—some researchers have proposed using exosomes as therapeutic agents. Since exosomes derived from normal T cells express TCRs, it is hypothesized that exosomes from CAR-T cells may similarly present chimeric antigen receptors, thereby replicating the functional properties of CAR-T cell receptors [65]. These exosomes offer several advantages over conventional CAR-T cells. Firstly, they can deliver granzyme and lysosomal enzymes to target cells without requiring perforin, either through direct fusion with the target cell membrane or via endocytic uptake [66]. Secondly, due to their small size, exosomes can traverse the biological barriers associated with tumors, allowing them to exert their effects on target cells [67]. Thirdly, exosomes can be loaded with antitumor drugs, significantly enhancing the efficacy of tumor-targeting therapies by delivering anticancer agents specifically to tumor cells [68]. Furthermore, exosomes have a propensity to engage with extracellular matrix of solid tumors, which further augments their therapeutic effectiveness [67].

### 3.4 CAR-T cell therapy

**Fig. 5:**
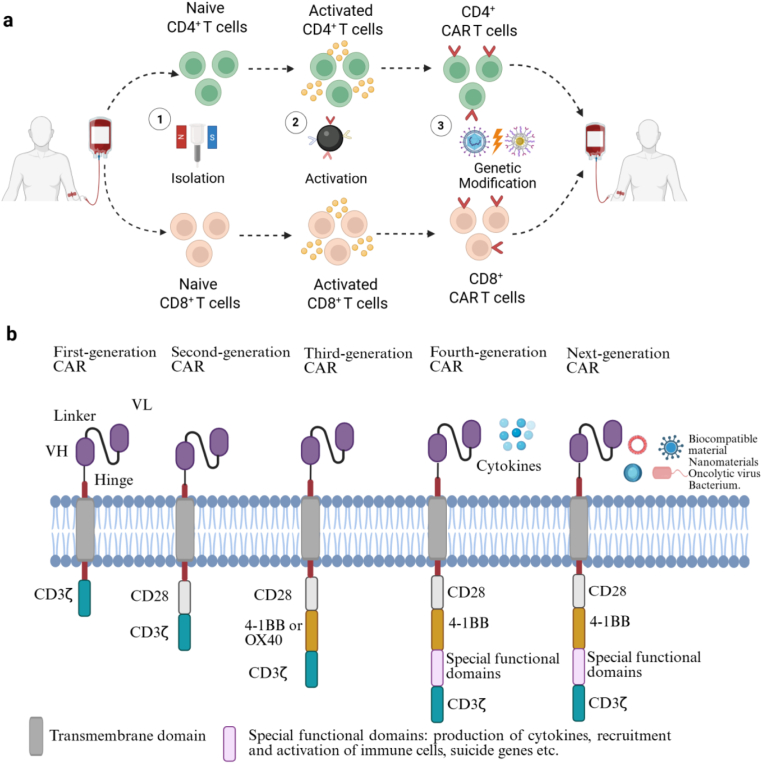
Schematic representation of CAR T cell manufacturing and generations**. a)** The manufacturing steps of CAR T cell, including apheresis, activation and genetic modification**. b)** Different generations of CAR T cell constructions. (www.BioRender.com.).

CAR-T cell therapy has arised as a ground-breaking advancement in the field of oncology, yielding exceptionally effective and sustained clinical outcomes. This innovative approach aims to reprogram T lymphocytes to target and eliminate neoplastic cells. The initial phase of this therapeutic process involves leukapheresis, which entails the extraction of a patient's peripheral blood. Apheresis is a commonly employed technique that facilitates the separation of blood into its constituent components, which are subsequently subjected to genetic modification before being reintroduced into the patient's system (Fig. 5a) [69]. CARs are artificially designed receptors that enable the redirection of lymphocytes, predominantly T cells, to identify and eradicate cells that express a specific target antigen. This interaction leads to robust T cell activation independently of MHC receptor and anti-tumor response. The anti-CD19 CAR-T cell therapy got approval from United States FDA in 2017 due to remarkable efficacy in treating B cell malignancies [70]. Since then, CAR-T cell therapy has been applied to various clinical conditions, including solid tumors [[71], [72], [73]], myocardial infarction [74,75], systemic lupus erythematosus [76,77], inflammatory bowel disease [78], lupus nephritis [79], and Sjögren's syndrome [80], among others. Multiple CAR-T cell therapies are currently under clinical trials for various diseases (see Table 1). CARs are modular synthetic receptors composed of an extracellular domain for binding target antigens, a transmembrane domain, a hinge region, and one or more intracellular signaling domains (Fig. 5b) [81]. Significant research efforts in CAR engineering have focused on elucidating the effects of CAR co-stimulation to develop CAR constructs with optimal endodomains. To date, upto five generations of CAR-T cells have been reported, encompassing various strategies aimed at improving the safety and efficacy of CAR-T cell therapies [82].

**Table 1:** CAR-T cell therapies under clinical trials for various pathological conditions.

NCT number	Title of study	Pathological condition	CAR targets	Location	Phase/status
**Cancer**					
NCT04464200	19(T2)28zixx Chimeric Antigen Receptor (CAR) T cells in People With B-Cell Cancer	Relapsed/refractory B-Cell Cancer	CD19	United States	Phase I (recruiting)
NCT06213636	Fourth-generation CAR T Cells Targeting CD 19/CD22 for Highly Resistance B-cell Lymphoma/Leukemia (PMBCL/CNS-BCL). (BAH241)	Relapsed/refractory B-cell acute lymphoblastic leukemia	CD19	China	Phase I (recruiting)
NCT03874897	Chimeric Antigen Receptor T Cells Targeting claudin18.2 in Solid Tumors	Advanced solid tumor with positive claudin	Claudin-18.2 CAR-T (autologous)	China	Phase I (recruiting)
NCT05420519	Clinical Study of CD70-targeted CAR-T Therapy for Advanced/​Advanced Renal Cancer	Advanced/metastatic renal cell carcinoma	CD70	China	Phase I (recruiting)
NCT04099797	C7R-GD2.CAR T Cells for Patients With GD2-expressing Brain Tumors (GAIL-B)	Neuroblastoma, glioma, medulloblastoma, or another rare brain cancer	GD2 CAR with C7R	United States	Phase I (active/recruiting)
NCT05239143	P-MUC1C-ALLO1 Allogeneic CAR-T Cells in the Treatment of Subjects With Advanced or Metastatic Solid Tumors	Ovarian, breast, pancreatic and other advanced or metastatic Solid Tumors	P-MUC1C-ALLO1 allogeneic CAR-T	United States	Phase I (recruiting)
NCT04995003	HER2 Chimeric Antigen Receptor (CAR) T Cells in Combination With Checkpoint Blockade in Patients With Advanced Sarcoma	HER2-positive breast and other cancers	HER2-CAR T	United States	Phase I (active/recruiting)
NCT03323944	CAR T Cell Immunotherapy for Pancreatic Cancer	Pancreatic Cancer	Mesothelin (Meso) CAR-T (autologous)	United States	Phase I (recruiting)
NCT05013372	CD147-CAR T Cells for Relapsed/​Refractory T Cell Non-Hodgkin's Lymphoma	Refractory T cell non-Hodgkin's lymphoma	CD147	China	Phase I (recruiting)
NCT06482905	Safety and Efficacy Study of TX103 CAR-T Cell Therapy for Recurrent or Progressive Grade 4 Glioma.	Progressive Grade 4 Glioma	B7-H3	United States	Phase I (recruiting)
**Autoimmune diseases**					
NCT06106906	A clinical study of CD 19 CAR-T in active systemic lupus erythematosus	Systemic lupus erythematosus (autoantibody -positive)	BCMA	China	Phase I (recruiting)
NCT05765006	CD19-CART(Relma-cel) for Moderate to severe active systemic lupus erythematosus	Systemic lupus erythematosus (refractory)	CD19	China	Phase I (completed)
NCT06384976	KYSA-7: A study of anti-CD19 CAR T-cells therapy, in subject with refractory primary and secondary progressive multiple sclerosis	Multiple sclerosis (progressive)	CD19	United States	Phase II (recruiting)
NCT06475495	Comparison of B-cell depletion by rituximab and anti-CD19 CAR-T therapy in patients with rheumatoid arthritis (COMPARE)	Rheumatoid arthritis (refractory)	CD19	Germany	Phase I/II (planned)
NCT06352281	Efficacy and safety of CAR-T cells therapy for chronic or refractory primary immune thrombocytopenia	Immune thrombocytopenia (refractory)	CD19	China	Phase I (planned)
NCT05859997	Universal CAR-T cells (BRL-301) in relapse or refractory autoimmune diseases	Necrotizing myopathy/systematic sclerosis	CD19 (allogeneic)	China	Phase I/II (active)
NCT06298019	Study of KYV-101 anti-CD19 CAR T therapy in adult dermatomyositis	Dermatomyositis (refractory)	CD19	United States	Phase I (recruiting)
NCT06428188	Sequential CAR-T cells targeting BCMA/CD19 in patients with relapsed/refractory autoimmune diseases (BAH247)	Lupus nephritis	BCMA	China	Phase I (recruiting)
NCT04422912	A phase 1/2, open-label, safety and dosing study of autologous CAR-T cells (DSG3-CAART) or CD-19 specific CAR-T cells (CABA-201), in subjects with active pemphigus vulgaris (RESET-PV)	*Pemphigus vulgaris*	CD19	United States	Phase I/II (recruiting)
NCT06585514	Anti-CD 19 chimeric antigen receptor T cells for SLE	SLE	CD19	China	Phase I/II (recruiting)
NCT04146051	Descartes-08 CAR-T cells in generalized myasthenia gravis (MG)	Myasthenia gravis (refractory)	BCMA	United States	Phase II (Active, not recruiting)
NCT06519565	Safety and efficacy of PRG-1801 in recurrent/refractory immune thrombocytopenia (ITP)	ITP (refractory)	BCMA	China	Phase I (recruiting)
NCT06675864	Open-label, multi-center, phase I/II study to assess safety, diseases progression, and cellular kinetics following YTB323 administration in participitants with non-active progressive multiple sclerosis	MS (progressive)	CD19	Multi-center	Phase I/II (active)
**Viral diseases**					
NCT03240328	The effect of CAR-T therapy on the reconstitution of HIV-specific immune function	HIV infection	HIV-1	China	Phase I (recruiting)
NCT03617198	CD4 CAR + ZFN-medified T cells in HIV therapy	HIV infection	HIV	United States	Phase I (active, not recruiting)
NCT04324996	A phase 1/2 study of universal off-the-shelf NKG2D-ACE2 CAR-NK cells for COVID-19 therapy	COVID-19	NKG2D-ACE2	China	Phase 1/2 (Unknown)
NCT04863066	Third-generation CAR-T cell therapy in individuals with HIV-1 infection (TCTIWHI)	HIV infection	HIV-1	China	Phase I (Unknown)
NCT03980691	Effect of chidamide combined with CAT-T or TCR-T cell therapy on HIV-1 latent reservoir	HIV infection	HIV-1	China	Phase I (completed)
NCT04648046	CAR-T cells for HIV infection	HIV infection	HIV-1	United States	Phase I/II (recruiting)

The manufacturing process for CAR-T cells is labor-intensive and protracted, imposing significant economic and physical burdens on patients. Initially, CAR-T cells are derived from lymphocytes isolated from peripheral blood, which are subsequently subjected to genetic engineering [83]. Current efficient genetic engineering techniques include gamma retroviral and lentiviral transfection; however, these methods are complicated by the substantial time and financial resources required for their intricate protocols. Non-viral methodologies, such as CRISPR-Cas technology and transposon-transposase systems, have also been extensively investigated for the introduction of CAR genes [84]. Nonetheless, the physical permeabilization of plasma membranes associated with these approaches can adversely affect cell viability, rendering them less suitable for widespread application. Additionally, neither electroporation nor viral-based methods can selectively transfect specific cell types from a heterogeneous population, necessitating a cell purification process prior to transfection [85].

Recent advances in gene delivery technologies have significantly enhanced the ability to engineer T cells in vivo for therapeutic applications. Innovations in viral vector design, alongside alternative nucleic acid nanodelivery systems such as polymer-based nanoparticle and liposomal nanoparticles, have substantially expanded the scope of achievable genomic modifications. These delivery platforms form a critical foundation for in situ T cell modification that simplify manufacturing processes and enables rapid therapeutic intervention for patients with progressive diseases [[86], [87], [88]]. This strategy involves the direct administration of nucleic acids to circulating T cells of patients with viral or non-viral vectors. The ribosomes then translate the delivered nucleic acid to express CARs or TCR on T cell surface. Notably, mRNA delivered with non-viral vector results in transient receptor expression, whereas delivery with viral vector provides more sustained expression. The clinical challenges associated with ex vivo T cell engineering, such as the need for lymphodepleting chemotherapy and its associated adverse effects can be overcome with help of in vivo T cell engineering. Furthermore, this technique may promote epitope spreading by preserving an intact immune system, thereby enhance a robust anti-tumor response. Alongside progress in ex vivo T cell engineering, in vivo modification has rapidly advanced from bench to bedside [89]. A pivotal study by Hunter et al. demonstrated the in vivo engineering of T cells for functional CAR in non-human primates via lipid nanoparticle (LNP)-mediated delivery. This approach achieved therapeutic efficacy in oncology and autoimmunity models, thereby establishing LNPs as a clinically viable platform for in situ T cell programming [86]. Similarly, LNPs have been shown to produce CAR-T cells in vivo both with and without antibody modifications [90,91].

## 4 T cell-inspired drug delivery to various diseases

### 4.1 Cancer

T cell-inspired drug delivery is an emerging and promising approach in cancer therapy, as T cells function as natural, antigen-specific carriers that can be harnessed to target tumor sites [92]. Utilizing T cells for targeted drug delivery represents a groundbreaking strategy to enhance the safety and efficacy of cancer treatments. This method allows for the effective use of the cytotoxic capabilities of these cellular carriers in parallel with the precise delivery of chemotherapeutic agents that combat tumors [93,94]. Considering the significant role of T cells, various strategies have been employed to activate or enhance T cell responses for cancer treatment. These strategies include CAR-T cell therapy to target the melanoma and synovial cell sarcoma. CAR-T cells or TCRs can recognize tumor-associated antigens, enabling the targeted killing of cancer cells. The CAR, first reported by Eshhar and his team was engineered to utilize the growth, cytotoxicity, and longevity of natural T cells while bypassing the MHC limitations of TCRs. CAR-T cells are modified to target overexpressed antigens on cancer cells. It is an experimental type of immunotherapy where patient's T-cells are modified to form CARs that recognize oncoantigens and enable the targeted killing of tumor cells. In liquid malignancies, where the CAR-T is already approved but in solid tumors, it is still mostly in clinical trials due to unique biological barriers. The various clinical trials are associated with tumor-associated antigens and no such antigen with similar properties to CD19 has been identified for CAR-T immunotherapy targeting solid tumors [[95], [96], [97], [98]]. This approach has been effectively utilized in treating different B cell malignancies and several therapies have been approved by U.S. FDA [99]. In 2017, the FDA approved CAR-T cell therapy targeting CD19 for the treatment of acute lymphoblastic leukemia [[100], [101], [102]]. Besides CD19, CD22, BCMA, CD20 and CD7 have been engineered with most clinical hematological malignancies for CAR-T therapy [[103], [104], [105], [106]]. The CAR-T cells developed from patients T-cells, and the CAR external domain binds the tumor-associated antigens, whereas the internal signaling domains activate the T cells to proliferate and release the cytokines and ultimately kill antigen-positive tumor cells. In case of solid tumors, the surface antigens that are highly expressed on tumor cells, including epidermal growth factor receptor (EGFR), human epidermal growth factor receptor 2 (HER2), carcinoembryonic antigen (CEA), mesothelin and mucin 1 (MUC1) [[107], [108], [109], [110]]. The EGFR are surface proteins enriched in pancreatic, colorectal, gastric, glioblastoma, and prostate cancers. The EGFR-targeted CAR-T therapy uses the CAR, and its external sensor identifies the EGFR on tumor cells, allowing the T-cells to bind and selectively kills EGFR-overexpressing tumors [[111], [112], [113], [114]]. HER2 is overexpressed in subsets of breast, gastric and other solid tumors. HER2 is overexpressed in mostly HER2-breast cancer and gastric cancers, whereas, the normal tissues express lower levels and rending it an ideal target for CAR-T cell therapy [[115], [116], [117]]. The CEA or CEACAM5 is a glycoprotein that is highly overexpressed in adenocarcinomas. It is considered as an oncofetal antigen, showing high expression level in fetal tissues and re-expression in tumor cells [118,119]. Mesothelin is a glycoprotein found on the surface of cells that is overexpressed in various solid tumors and is commonly used as the target for CAR-T cell therapy in malignant mesothelioma, lung cancers, ovarian cancer and pancreatic duct adenocarcinoma. The high and relatively tumor-specific expression, mesothelin also involves in tumor adhesion and invasion and making an attractive for engineered T cells [120,121]. MUC1 is a cell surface glycoprotein that is overexpressed in many epithelial cancers. The elevated level of MUC1 is involved in regulation of various signaling pathways and plays a crucial role in tumor cell metabolism, programmed cell death, epithelial mesenchymal transition, and distant metastasis [[122], [123], [124]]. CAR-T cells are mostly used to identify particular antigens on cancer cells and allow them to target tumors selectively. Using the patient's own T cells lowers the chances of complications and provides better therapeutic efficiency for treatment of various types of cancers. They also serves as a potential for long-term immune surveillance and studies reported have shown that many patients have improved quality of life. Li et al. fabricated the HER2-targeted CAR-T cells and evaluated their anti-tumor effects on glioblastoma. The potential side effects were monitored when targeting the HER2 receptors that were overexpressed in glioblastoma. The HER2-CAR-T showed cytokines secretion and strong toxicity against glioblastoma. Moreover, the therapeutic improvement was enhanced when delivered by peritumoral injections. The HER2-CAR-T cells showed high anti-tumor efficacy against glioblastoma, indicating a potential CAR-T cell treatment strategy [125]. Folmont et al. reported the mesothelin-targeted CAR-T therapy for prostate cancer. The mesothelin expression level across various preclinical prostate cancer models and its therapeutic efficacy were evaluated. The mesothelin-targeted CAR-T therapy depicts enhanced cytotoxicity and showed selective targeting towards tumor cells under both hypoxia and normoxia. The mesothelin-targeted CAR-T therapy displayed potential strategy for targeting prostate cancer [126]. Zhang et al. reported the T-cell enhancing scaffolds that were subcutaneously injected to form a tumor microenvironment promoting T-cell infiltration, restimulation and recruitment. The T-cell ligands (anti-CD3 and anti-CD28) were activated, and the T-cell enhancing scaffolds were found to drive the CAR-T cell proliferation, differentiation and cytotoxic activity, ultimately enhancing the anti-tumor efficacy of the cells [127] Moreover, T cells can carry various types of nanoparticles loaded with chemotherapeutic agents directly to tumors, maximizing the therapeutic cargo concentration while minimizing the systemic side effects [128,129].

**Fig. 6:**
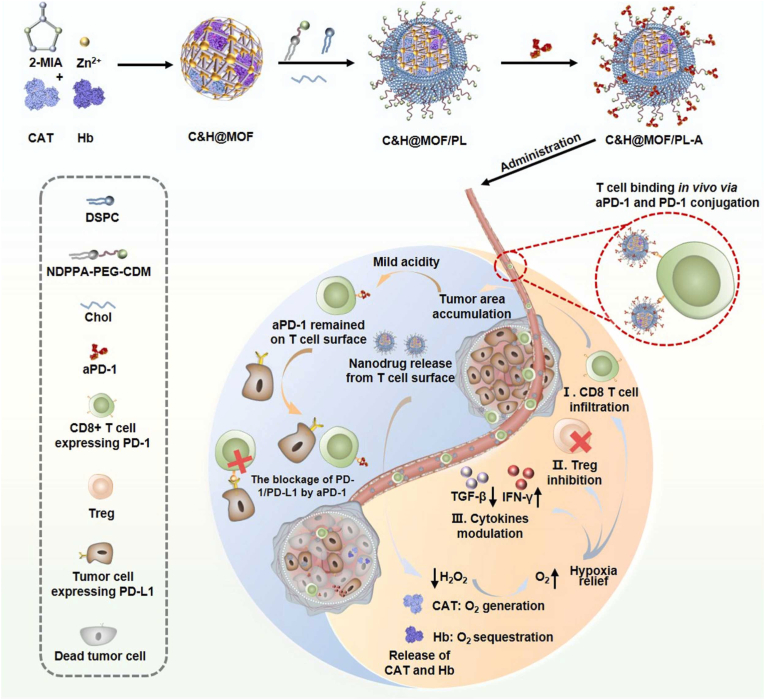
Schematic representation of synthesis and therapeutic mechanism of nanomedicine (C&H@MOF/PL-A). C&H@MOF was synthesized by encapsulating catalase and haemoglobin into MOF. The C&H@MOF/PL was obtained by coating C&H@MOF with functional liposome to improve stability, and for T cell binding, aPD-1 was attached onto surface of C&H@MOF/PL to obtain nanomedicine. Adopted from Ref. [38]. Copyright 2024, Elsevier.

T cell hitchhiking represents a novel technique that exploits the natural ability of T lymphocytes to target the cancer cells and infiltrate tissues, facilitating the direct transport of therapeutic agents to the tumor microenvironment. This approach enhances the precision and effectiveness of therapy by attaching drug-loaded nanomaterials or other therapeutic cargo to T cells. T cell hitchhiking offers prolonged circulation, improved tumor-targeting specificity, and reduced off-site toxicities [34,130]. Huang et al. [38] showed pH-responsive, T cell-targeting MOF nanoparticles for the co-delivery of hemoglobin, catalase, and anti-PD-1 antibody to synergistically modulate the immunosuppressive tumor microenvironment, as shown in Fig. 6. The acidic pH of the tumor microenvironment causes the nanoparticles to detach from T cells, allowing the PD-1 antibody to bind to T cells and enable immune checkpoint blockade therapy of colorectal cancer. This study heralds a new era of immunotherapy, characterized by the synergistic combination of hypoxia modulation and immune checkpoint blockade, and holds promise for redefining the parameters of effective colorectal cancer therapy. T cell hitchhiking-mediated drug delivery has been utilized for the photothermal therapy of tumors. Due to minimal side effects to healthy tissues, photothermal therapy are considered alternative approach to cancer chemotherapy. This technique involves photothermal stimulation that enhance the expression of heat shock protein in tumor microenvironment. Yang et al. [131] reported the biomimetic aggregation-induced emission (AIE) nanoparticles for T-cell hitchhiking cancer targeting and NIR-II fluorescence-based photothermal therapy. The AIE containing photothermal agents with hitchhiking ability were fabricated by coating DC membrane on nanoaggregates of the NIR AIE polymeric photothermal agents. The enhanced tumor delivery efficiency and hitchhiking technique showed the effective delivery of therapeutic cargo in cancer photothermal immunotherapy.

The innovative strategy of using T cell membrane-coated bionic nanoparticles leverages the natural targeting and immune-evasive functions of T-cell membranes to enhance the delivery of chemotherapeutic agents to the tumor microenvironment. Beyond their role as efficient drug delivery systems, T cell membrane-coated nanoparticles improve targeting and allow for diverse therapeutic applications [13,132]. T-cell membrane-coated nanoparticles have significantly improved outcomes in cancer immunotherapy. Recent research showed that aberrant activation of immune checkpoint blockades, such as LAG-3, amplifies immunosuppressive signaling by associating with the TCR-CD3 complexes. The FGL-1 is overexpressed in tumors and ultimately drives T-cell exhaustion via the LAG-3 signaling pathway. T-cell membrane-coated nanoparticles overcome the anti-PD-1 resistance by remodeling the immunosupressive tumor microenvireonment and targeting of FGL-1/LAG-3 pathway [133]. Besides the immunotherapy, the photothermal therapy also causes immunogenic cell death in tumor tissues and consider as a synergistic mechanism for bladder cancer immunotherapy. Deng et al. [134] fabricated bionic T cell membrane-coated nanoparticles for synergistic photothermal-immunotherapy for bladder cancer by triggering cuproptosis. The Tim-3 overexpressing T cell membrane-coated nanoparticles enhance the bladder cancer immunotherapy. The overexpressed Tim-3 in the T-cell membrane-coated cells depicts better tumor targeting ability and enables them to be recognized by Galectin-9 on tumor cells. Lei et al. developed the CAR-derived cell membrane-coated nanoparticles for delivering an mRNA-based cancer gene therapy. The CT26 cells expressing a HER2-specific CAR molecules were developed to extract the CAR-CT26 membrane. These nanoparticles depicts enhanced mRNA transfection efficiency towards CT26 cells overexpressing HER2 antigens, enhanced tumor targeting and suppression [135]. Moreover, Wang et al. also reported a local photothermal therapy for glioblastoma recurrence. The fabricated T-cell biomimetic nanoparticles having aggregation-induced-emission active luminogens were used as NIR-II photothermal therapeutic agents. The T-cell biomimetic nanoparticles are used for CD133 and EGFR, which are overexpressed in glioblastoma cells [136]. Various studies reported that the use of polymeric nanoparticles camouflaged with human cytotoxic T-lymphocytes in combination with low-dose radiation therapy for treatment of gastric cancer. These biomimetic nanoparticles maintained prolonged circulation time and tumor-targeting specificity of cytotoxic T-lymphocytes, while the localized low-dose irradiation significantly enhanced tumor localization [137].

Exosomes, a type of extracellular vesicle, play a critical role in intercellular communication within the tumor microenvironment, thereby modulating the immune response to cancer therapy [138]. These nano-sized vesicles are secreted by various type of cells, including T cells. T cell-derived exosomes retain the characteristic of their progenitor T cells, carrying surface proteins, RNA and various signaling molecules that influence target cells. T cells are essential components of adaptive immunity, and T cell-derived exosomes are interested area of investigation for researchers [[139], [140], [141]]. T cell-derived exosomes can activate various immune cells, modulate immune responses, and contribute to antigen presentations [142]. They also directly transfer cytotoxic molecules, such as Fas ligand and granzyme B, to tumor cells, inducing apoptosis. Additionally, they modulate immune cells by activating effector T cells, regulating immune checkpoints, and facilitating antigen presentation [143,144]. T cell-derived exosomes can enhance tumor elimination by disrupting the PD-1/PD-L1 pathway. The T cell receptor signaling pathways and cell-binding mechanism can increased expression in exosomes overexpressing PD-1 as compared to microvesicles. These PD-1 expressing exosomes significantly enhanced the proliferation and activity of CD8^+^ T cells within the tumor microenvironment. They have the potential to counteract the immunosuppressive microenvironment by inhibiting the PD-1/PD-L1 interaction and promoting apoptosis in tumor cells via Fas ligand and granzyme B [145]. Various studies have demonstrated that exosomes derived from various sources, such as Tregs [64,146], helperT cells [60,147,148], and cytotoxic T cells [149,150] play a role in tumor immune modulation. The T-cell-derived exosomes are considered as genetically engineered modified extracellular vesicles that serve as a novel nanocarriers due to their tumor targeting efficacy. Zhu et al. [66] reported the hybrid nanovesicle for lung cancer immunotherapy. These nanovesicles were formed by fusing lung targeted liposomes with CAR-T cell-derived exosomes, targeting both mesothelin and PD-L1. The nanovesicles deliver the chemotherapeutic agent to tumor cells through sequential targeted delivery and enhanced anti-tumor effects.

### 4.2 Infectious diseases

Infectious diseases represent a major global health challenge. The escalating threat of antimicrobial resistance demands an urgent need for alternative therapeutic strategies [151]. T cell-mediated drug delivery is emerging as a novel approach for treating infectious diseases, particularly those caused by chronic or intracellular pathogens that evade conventional therapies. T cells naturally home to sites of infection and inflammation, making them ideal “living carriers” for delivering therapeutic agents with high specificity. Unlike synthetic nanocarriers, T cells can circulate continuously, transmigrate across biological barriers, and localize within pathogen reservoirs such as lymph nodes, granulomas, or tissues harboring latent infections. Therapeutic payloads—including antiviral drugs, antimicrobial peptides, cytokines, or nanoparticles—can be attached to T cell membranes or delivered via engineered exosomes. Furthermore, genetic modification of T cells, such as the development of pathogen-specific CAR T cells, enhances their ability to recognize and target infected cells. This approach not only ensures localized drug release but also modulates T cell cytotoxicity to directly eliminate infected reservoirs [152]. CAR T cell therapy is a promising novel treatment modality, initially developed for cancer but increasingly applied to chronic infectious diseases. CAR T cell therapy has garnered considerable interest, with numerous efforts aimed at developing treatments for patients suffering from chronic viral infections. To date, several CAR constructs targeting infectious diseases have been identified, including those for HIV, cytomegalovirus (CMV), hepatitis B virus (HBV), and hepatitis C virus (HCV). These therapies have made significant progress and are currently undergoing clinical evaluation (Table 1) [153]. The application of CAR-T therapy in HIV has evolved substantially, progressing from early CD4-based constructs to more sophisticated designs incorporating broadly neutralizing antibodies (bNAbs) and dual-specific targeting strategies. Early studies by Deeks et al. demonstrated the potential of CD4^+^ CAR T cells to reduce viral reservoirs. Similarly, Leibman et al. emphasized the critical role of optimized vector design and costimulatory domains in enhancing antiviral efficacy [[154], [155], [156]]. Moreover, Ali et al. and Liu et al. have highlighted the potential of bNAb-based CAR-T cells in controlling viral rebound and specifically targeting HIV-infected cells. The development of bispecific CAR-T cells, as reported by Liu et al., along with carbohydrate recognition domain–based CAR-Ts, exemplifies how multi-targeting strategies can enhance therapeutic potency while reducing immunogenicity. Collectively, these advances underscore that anti-HIV CAR-T therapy, although still facing challenges in vivo, is a rapidly evolving field with the potential to transform HIV management by targeting both active and latent reservoirs [153,157,158]. HBV infection remains incurable. While antiviral agents suppress HBV replication, they do not eradicate the infection. A robust effector T-cell response is necessary to eliminate HBV, but this response is typically absent in patients with chronic infection. Consequently, patients with chronic HBV often fail to develop durable immune responses, increasing the risk of liver cancer. To enhance immune control, Krebs et al. developed S-CAR-T cells targeting the three HBV envelope proteins (S, M, and L), which primarily reduced viral DNA and HBV-infected hepatocytes but were limited by T-cell exhaustion [159]. Similarly, Kruse et al. engineered anti-HBs-G4m CAR-T cells that effectively recognized HBsAg particles and HBV-positive hepatocytes in vitro and significantly decreased HBV DNA and antigen levels in vivo. Together, these studies highlight the therapeutic potential of CAR-T strategies for HBV, although overcoming T-cell exhaustion and host immune responses remains a critical challenge [159,160]. In 2014, Kumaresan et al. developed a glycan-specific CAR by fusing the extracellular domain of the human Dectin-1 receptor for β-glucans to the transmembrane CD28, along with the CD3ζ signaling domain. This Dectin-1 CAR effectively recognized Aspergillus hyphae and demonstrated strong antifungal activity both in vitro and in vivo, significantly restricting fungal growth [161]. The same group later extended this concept in 2020 to target Cryptococcus spp., which cause over one million infections annually. In this iteration, the Dectin-1 domain was replaced with a single-chain variable fragment (scFv) specific for glucuronoxylomannan (GXM), the primary cryptococcal capsular antigen. This GXM-CAR T cell exhibited high specificity and potent activity against cryptococcal infections in preclinical studies [162]. SARS-CoV-2 has had profound global impacts, overwhelming healthcare systems, disrupting economies, and altering social structures. At the onset of the pandemic, no effective therapy was available for severe COVID-19 disease. Ma et al. generated a novel CAR-NK cells for targeting SARS-CoV-2. These CAR-NK cells were engineered using the scFv domain of CR3022, a broadly neutralizing antibody against both SARS-CoV-1 and SARS-CoV-2. CR3022-CAR-NK cells specifically bind to the receptor-binding domain of SARS-CoV-2 and to pseudotyped SARS-CoV-2 spike (S) protein. They can also be activated by pseudotyped SARS-CoV-2 S viral particles in vitro. The development of COVID-19 treatments using CAR-NK cell therapy, alongside other antibody-based therapies, offers minimal toxicity and high efficacy, presenting a promising option for patients with severe COVID-19 disease [163].

T-cell hitchhiking refers to a novel drug delivery strategy in which therapeutic cargos or nanoparticles are cloaked with or attached to T cells or their membranes, thereby exploiting their natural homing ability to sites of inflammation or infection. This approach prolongs systemic circulation, enhances targeted delivery, and facilitates immune evasion while maintaining therapeutic efficacy. Recent advances in immune cell membrane-coated nanoparticles, including T-cell-derived systems, demonstrate potential applications in infectious diseases by improving antimicrobial drug localization, neutralizing bacterial toxins, and enhancing vaccine responses [164]. Murooka et al. provided compelling in vivo evidence that HIV exploits the natural migratory capacity of CD4^+^ T cells to promote viral spread. Using intravital two-photon microscopy in humanized mice, they observed that HIV-infected T cells retained motility, enabling local dissemination within lymphoid tissues through tethering interactions that may facilitate cell-to-cell transmission via virological synapses. More importantly, these infected T cells acted as vehicles for systemic dissemination through physiological T-cell recirculation, a process shown to be essential for the establishment and maintenance of plasma viremia. These findings not only reveal how pathogens exploit host immune dynamics but also underscore the potential benefits of harnessing the same “T-cell hitchhiking” phenomenon for therapeutic drug delivery. By engineering T cells as mobile carriers, therapeutic agents can be directed precisely to infected reservoirs, offering a novel and more effective approach to combat persistent infections such as HIV [165].

**Fig. 7:**
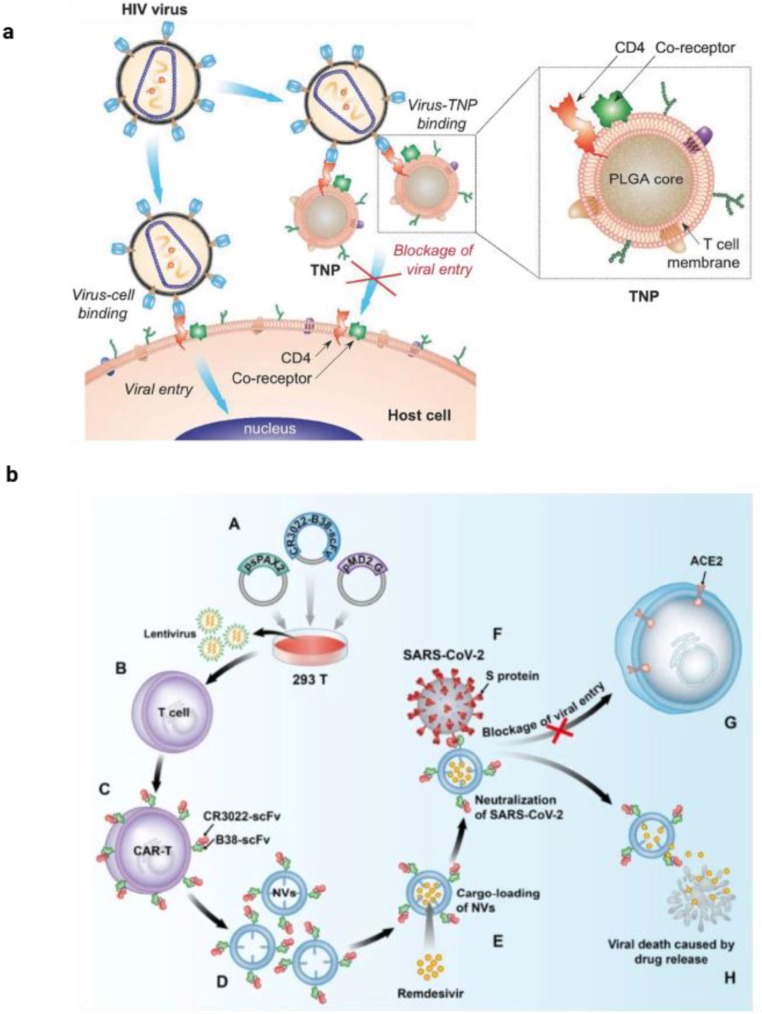
a. Schematic illustration of T cell-membrane-coated nanoparticles (denoted as TNPs) aimed for diminishing HIV infectivity. These TNPs were designed by covering PLGA core with natural CD4^+^ T-cell membranes that exhibit essential antigens, having CD4 receptor and CXCR4 or CCR5 co receptors for specific viral targeting. By simulating the surface antigen profile of native T cells, the TNPs inhibit the entry of HIV viruses and thus prevent the infection host target cells. Adopted from Ref. [168]. Copyright 2018, John Wiley & Sons. **b)** illustration of bispecific CAR-T cells-derived nanovesicles targeting the spike proteins of SARS-CoV-2 for treating COVID-19. Adopted from Ref. [169]. Copyright 2021, Springer nature.

To date, nanoparticles have been extensively explored for the treatment of various diseases. However, their applications are limited by challenges such as non-specificity, poor biological stability, rapid clearance, and recognition as foreign substances. To overcome these issues, bionic cell membrane coating technology has emerged as a novel strategy for nanoparticle design. This approach has gained significant attention due to its biocompatibility and targeting capabilities [166]. Coating nanocarriers with T cell membranes imparts intrinsic immunological functionalities, including cytokine receptor expression, antigen recognition, and lymphoid tissue trafficking, thereby facilitating precise spatiotemporal drug delivery. This biomimetic strategy not only enhances immune evasion and circulation stability but also exploits T cell-specific homing mechanisms to improve therapeutic selectivity in infectious and inflammatory diseases [167]. Wei et al. developed CD4^+^ T cell membrane-coated PLGA nanoparticles (TNPs) that retain the functions and intrinsic biomarkers of T cells. Specifically, the TNPs are enriched with CD4 and CCR5/CXCR4 receptors. Using two distinct HIV strains, X4 and R5, they demonstrated that the TNPs selectively bind to the HIV envelope glycoprotein gp120 and effectively inhibit gp120-induced apoptosis of CD4^+^ T cells (see Fig. 7a). Furthermore, the TNPs successfully neutralized both HIV strains, thereby preventing viral infection of peripheral blood mononuclear cells. Overall, these CD4^+^ T cell membrane-coated TNPs represent a promising and innovative approach for HIV treatment [168].

T cell-derived exosomes represent an innovative drug delivery system that harnesses the natural immunological communication mechanisms of T cells, offering intrinsic targeting of infected or inflamed tissues. Their biocompatibility, nanoscale size, and ability to carry bioactive molecules—including drugs, nucleic acids, and proteins—make them highly suitable for precision therapy in infectious diseases, where conventional carriers often face limitations. These exosomes are a crucial component of adaptive immunity, and their functions have been extensively studied in the context of various infectious diseases, autoimmunity, and inflammatory responses [167]. Given the threat posed by the COVID-19, effective treatment was required to cope with the pandemic. Small-molecule drugs such as remedsvir and neutralizing antibodies was considered the most promising treatment for this infection. However, several potential issues must be addressed, including toxic effects of remedsvir, viral resistance to antibody-mediated neutralization, and the unexpected antibody-dependent enhancement effect. As an alternative, Zhu and colleagues developed a novel type of programmed nanovesicle derived from bispecific CAR-T cells. Using T cells as a model, they created nanovesicles capable of neutralizing viruses and delivering drugs. These nanovesicles display two scFvs, named B38 and CR3022 (see Fig. 7b). CR3022 targets conserved epitopes on the spike protein of both SARS-CoV-1 and SARS-CoV-2, while B38 binds to the receptor-binding domain of SARS-CoV-2, blocking the virus from attaching to the ACE2 receptor [169].

### 4.3 Autoimmune diseases

Autoimmune diseases are dysregulated immune responses, representing a significant factor in the morbidity and mortality associated with chronic diseases. The abnormal immune activation of the immune system leads to the generation of autoreactive T cells that target and attack self-tissues [[170], [171], [172], [173], [174]]. Targeting autoreactive T cells or their pathological niche using T cell-mediated drug delivery can facilitate selective immune modulation, thereby lowering the risks of systemic immunosuppression. T cell-mediated drug delivery in autoimmune diseases represents a novel therapeutic approach that utilizes the properties of T cells to selectively target and modulate the pathogenic immune response underlying autoimmunity [[175], [176], [177]]. By harnessing the inherent characteristics of T cells and modifying them or their components to modulate autoreactive immune responses accurately, these therapies aim to achieve better therapeutic efficacy with less side effects than traditional immunosuppressant [138]. Different types of engineered T cells can be used to deliver therapeutic agents selectively to autoreactive immune cell populations, such as B cells that produce autoantibodies. In most autoimmune diseases, autoreactive T and B cell clones, as well as autoantibodies targeting a patient's own antigens, are formed before the symptoms appear [178].

The various receptors that are overexpressed in autoimmune diseases includes Toll-like receptors (TLRs) that is pattern recognition receptors (PRRs) critical in both innate and adaptive immunity [179,180]. Besides TLRs, the NOD-like receptors (NLRs) also play important roles in regulation and antigen presentation. The NLRs are subdivided into five families including NLRA, NLRB, NLRC, NLRP and NLRX. Among the various types of autoimmune diseases, the NOD1 and NOD2 are overexpressed in rheumatoid arthritis (RA). These receptors initiates the production of inflammatory cytokines that ultimately results in the pro-inflammatory biological effects [181]. The B cell also plays a crucial role in the immune response and ultimately leads to autoimmune diseases. The B cell receptors (BCRs) also plays an important role in some autoimmune diseases, like SLE [182,183]. There are various receptors that features overexpression of the various receptor families on immune, synovial and endothelial cells. The overexpression can drive chronic inflammation, joint destruction in RA. These receptors include chemokine receptors [184,185], toll-like receptors [186,187], cytokine receptors [188], and B cell activating factor receptor [189,190]. The various T cell-mediated drug delivery methods include CAR T cells, T cell hitchhiking, T cell membrane coating, and T cell-derived exosomes can be harnessed to target these receptors for the treatment of autoimmune diseases.

**Fig. 8:**
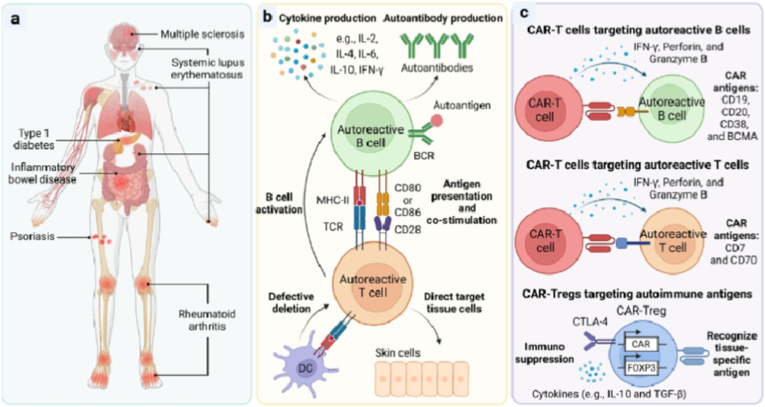
Role of autoreactive B and T cells in autoimmunes diseases and mechanism of CAR-T cells in treatment of autoimmune diseases. **a)** various types of autoimmune diseases. **b)** Role and interactions of autoreactive B and T cells in initiating autoimmunes diseases. **c)** Development of CAR-t cells targeting autireactive B and T cells and CAR-Tregs targeting autoimmune antigens. Adopted from Ref. [194]. Copyright 2024, Cell Press.

The CARs are the engineered receptors that alter the activity and specificity of T cells and various immune cells. This can be achieved by combining the monoclonal antibodies with the intracellular signaling components of the TCR. The CAR-T cells have gained significant attention in the field of cancer, prompting further research into its applicability for the treatment of other diseases. The approach of CAR-T therapy for autoimmune diseases has been adapted since 2021 to reset the immune response in various autoimmune diseases where standard immunosuppressive agents fail, leading to rapid symptomatic relief and potential long-term effects [191,192]. Therefore, CAR-T cells is widely utilized to target and eliminate autoreactive immune cells that contribute to autoimmune diseases like SLE, psoriasis, multiple sclerosis (MS), RA, and Crohn's disease [86,178,183,[193], [194], [195], [196], [197], [198], [199], [200]] (Fig. 8).

The CAR-T cells used to treat the autoimmune diseases carry a chimeric antigen receptor that targets specific proteins on B cells, such as CD19, and triggering their destruction and preventing the production of autoantibodies. CAR-T therapy offers several key advantages for autoimmune diseases such as targeted and potent immunomodulation, reduced side effects and improved safety. The various types of receptors are overexpressed in autoimmune diseases and its possible CAR-T therapy represents a paradigm shift in autoimmune disease management and improved quality of life [192]. Among the various diseases, MS is characterized by an autoimmune and neurodegenerative disorder and the key autoantigens targeted by Th1/Th17, B cells and CD8^+^ are myelin basic protein and myelin oligodendrocyte glycoprotein. Yi et al. [201] engineered the CAR-T cells therapy to recognize and kills autoantigen by peptide-MHC II domain for the treatment of MS. The peptide-MHC II CAR-T cells prevented demyelination in an experimental autoimmune encephalomyelitis (EAE) model. Moreover, in , the various receptors that have been targeted are CD19, CD20, B-cell maturation antigen (BCMA). The CAR-T therapy for RA have been widely used to recognize these receptors, leading to the killing of B cells. The therapy is more effective where autoantibodies play a major role in disease progression [202,203]. Li et al. [204] reported CAR-T cell therapy for RA, targeting CD19 receptors that was overexpressed on most B-cells. The CD19 B-cells were completely eliminated, clearance of RA-related autoantibodies and sustained clinical response in RA patients. A study by Jin et al. [205] showed the efficacy of anti-CD19 CAR-T cells for the treatment of SLE. The transfer of anti-CD19 CAR-T cells not only prevented the onset of disease symptoms but also provided the therapeutic effects at a later stage following the SLE progression. Likewise, the anti-CD19 CAR T cells in SLE patients were also reported, depicting that CD19 CAR T cells transfer is feasible and effective in SLE treatment [77].

The strategy of utilizing T cells as carrier for drug delivery in autoimmune diseases holds a promising approach that uses the natural ability of T cells, aiming for precise treatment with improved safety and effectiveness. The therapeutic moiety are delivered or attached via these T cells that have the ability to migrate and infiltrate the disease tissues in autoimmune diseases. T cell-mediated drug delivery strategy employs engineered T cells to precisely recognize and modulate pathogenic immune cells such as autoreactive B or T cells, by leveraging their ability to migrate and interact with other immune cells. This strategy minimizes the systemic immunosuppression and exploits the natural T cell trafficking and homing to inflamed or lymphoid tissues. The conventional approaches that suppress the immune system, hitchhiking strategies aim to deliver the immunomodulatory agents, gene therapy or cytotoxic agents, aiming for precise treatment, improved safety and efficacy. In T-cell hitchhiking therapy, the T-cells or bispecific antibodies attach as a “hitch a ride” on the immune cells, aiming to deliver payloads or regulate their function by interacting with immune checkpoints [34,206,207].

Among the various surface-functionalized approaches for autoimmune diseases, the cell membrane coated approach has gained significant attention in improving the T-cell mediated drug delivery to cell therapy. T-cell membrane-coated or biomimetic therapies for autoimmune diseases utilize the nanoparticles or various cells that are camouflaged in natural T-cell membrane to cloak the behaviour of T-cells. The T-cell biomimetic effect enables the targeted delivery and modulation of immune response. These biomimetic nanoparticles utilize the surface proteins and receptors that are found on T-cells and enable them to avoid immune clearance, enhanced systemic circulation and delivery of therapeutic cargo to site of action. In autoimmune diseases, using biomimetic T-cell membrane therapies can carry immunosuppressants to immune cells and thereby reducing the inflammation and tissue damage. This approach can also be engineered to carry various types of antigens or antibodies on their cell surface and enabling them to target the depletion of autoreactive B or T cells and modulation of immune response in various diseases like SLE, and RA [200,208,209]. The various receptors that are leveraged in biomimetic approach for various autoimmune disease include T-cell receptor (TCR), immune checkpoint receptor like PD-1, CD3 and CD4 [[210], [211], [212], [213]].

In autoimmune diseases, T-cell derived exosomes are small extracellular vesicles secreted by T cells plays a pivotal role in immune response. These exosomes can carry proteins, nucleic acid moiety and can mediate cell-to-cell interaction by transferring bioactive molecules to the various cells. T-cell derived exosomes can carry immunosuppressant including TGF-B and CTLA-4 that plays an important role in regulation of immune response. The various immunomodulatory surface markers such as CD73 and CD25 have been identified within the T-cell derived exosomes [214,215]. Azimi et al. [216] reported the immunomodulatory effects of exosomes derived from regulatory T-cell for the therapy of MS. CD63^+^ exosomes were isolated from regulatory T cells and then co-cultured with conventional T cells. The results advocate that regulatory T-cells derived exosomes suppressed T-cell proliferation in multiple sclerotic patients. The T-cell derived exosomes can regulate both immune cell activation and apoptosis. The engineered exosomes can deliver immunosuppressive molecules such as PD-1 or Fas ligand (FasL) to promote the immune tolerance and reduce inflammation. Therefore, T-cell-derived exosomes are key players in both the regulation and pathogenesis of autoimmune diseases. The capability to deliver the regulatory molecules and modulate immune cell function makes them an ideal target for cell-based therapies in autoimmune diseases.

### 4.4 Cardiovascular diseases (CVDs)

CVDs remain one of the leading causes of death worldwide, accounting for 18.6 million deaths in 2019, which represents one-third of all global deaths. Of these, 10.8 million CVD deaths were attributed solely to high systolic blood pressure, resulting in 235 million disability-adjusted life years (DALYs). Other significant contributors include dietary risks (6.1 million deaths), high LDL cholesterol (4.4 million deaths), and air pollution (3.0 million deaths). Despite substantial advances in prevention and treatment, over 10 million premature CVD deaths annually could be prevented through effective risk factor control. The burden is disproportionately higher in low- and middle-income countries, which account for more than 75% of global CVD mortality. Therefore, intensified strategies focusing on hypertension control, lipid management, obesity reduction, and tobacco cessation, combined with stronger health systems and equitable global policies, are urgently needed to curb this epidemic [217].

T cell-inspired drug delivery offers an innovative therapeutic approach for CVDs by leveraging the natural homing and immune recognition capabilities of T cells to specifically target fibrotic or inflamed cardiac tissue. Engineered T cells, primarily CAR-T cells, can be directed against activated cardiac fibroblasts, thereby reducing fibrosis and remodeling while improving cardiac function. This system combines targeted therapeutic delivery with immune specificity, providing a long-lasting, precise, and low-toxicity treatment compared to conventional drug delivery methods. Collectively, these strategies have established T cell-mediated therapies as a successful and novel approach for managing cardiac diseases [218]. Rurik et al. developed T cell–targeted LNPs to transiently generate antifibrotic CAR-T cells by delivering modified mRNA. The efficacy of this system was evaluated by injecting CD5-targeted LNPs into a heart failure mouse model. The modified mRNA encoding the CAR was efficiently delivered to T lymphocytes, resulting in the transient production of functional CAR T cells in vivo. These antifibrotic CAR T cells exhibited trogocytosis and engaged their target antigen as they accumulated in the spleen. Thus, the developed mRNA-targeted LNPs represent an effective and promising platform for treating various cardiac conditions and diseases [75]. Atherosclerosis is responsible for 18 million deaths each year, underscoring the urgent need for novel medical therapies, particularly for patients ineligible for surgical interventions. Building on this, Schwab et al. employed an inducible Treg cell strategy by developing an anti-oxidized low-density lipoprotein (OxLDL)-specific CAR Treg treatment that produces immunosuppression through both cell- and cytokine-mediated mechanisms, thereby reducing the formation of macrophage foam cells. In immunocompetent mouse models, the anti-OxLDL CAR Tregs inhibited approximately 80% of plaque formation associated with atherosclerosis and hyperlipidemia. This system demonstrates a novel therapeutic strategy to prevent plaque deposition and inflammation linked to OxLDL in atherosclerosis [219].

T cell hitchhiking presents a novel therapeutic strategy for CVDs by leveraging the natural homing ability of T lymphocytes to inflamed or injured cardiac tissue. Drug-loaded nanocarriers that utilize T cell hitchhiking can circulate longer in the bloodstream, evade rapid clearance, and specifically accumulate at sites of atherosclerotic lesions or myocardial injury. This approach not only enhances site-specific drug delivery but also reduces systemic toxicity, representing a promising advancement over conventional delivery systems [220]. Cardiac fibrosis is characterized by excessive deposition of extracellular matrix proteins, leading to impaired ventricular compliance and myocardial stiffening. This pathological remodeling contributes to the progression of heart failure, arrhythmias, and poor outcomes in various cardiovascular diseases. Aghajanian et al. demonstrated the efficacy of redirected T cell immunotherapy to precisely target pathological cardiac fibrosis in mice. Their findings indicate that cardiac fibroblasts expressing a xenogeneic antigen can be effectively targeted and eliminated through the adoptive transfer of antigen-specific CD8^+^ T cells. Specifically, the adoptive transfer of T cells engineered with a CAR targeting fibroblast activation protein significantly reduces cardiac fibrosis and restores heart function following injury in mice. Overall, the study shows that adoptive transfer of CAR-engineered T cells leads to a substantial reduction in cardiac fibrosis and improvement in cardiac function [221].

T cell membrane-coated drug delivery systems offer a biomimetic strategy for treating CVDs by leveraging the natural homing ability and immune-interactive properties of T cells. These systems can extend circulation time, prevent rapid clearance, and enable targeted delivery to injured or inflamed cardiovascular tissues, thereby reducing off-target toxicity and enhancing therapeutic accumulation. Furthermore, the T cell membrane provides functional adhesion molecules and receptors that facilitate precise navigation to diseased vascular sites, overcoming the limitations of conventional nanocarriers. This innovative approach represents a novel and promising platform with significant potential for precision cardiovascular therapeutics [222]. Xiong et al. reported the development of macrophage–T cell hybrid membrane-camouflaged biomimetic nanoparticles encapsulating IRF1-siRNA, demonstrating significant efficacy in suppressing macrophage pyroptosis and ameliorating autoimmune myocarditis. The hybrid membrane coating provided enhanced targeting of inflamed myocardial tissue and pro-inflammatory macrophages, enabling effective siRNA delivery with minimal off-target effects. This developed system thus offers a novel and safe therapeutic approach for cardiac diseases, providing targeted intervention with improved therapeutic precision and biocompatibility [50].

T cell–derived exosomes have emerged as novel modulators in CVDs by transferring immunoregulatory miRNAs and proteins that influence inflammation, fibrosis, and vascular remodeling. They regulate key processes such as fibrosis, inflammation, cardiomyocyte survival, and angiogenesis, thereby affecting the progression of atherosclerosis and myocardial repair. Due to their low immunogenicity, nanoscale size, and biocompatibility, exosomes offer greater advantages over cell-based therapies for targeted drug delivery. Their dual role as both disease mediators and therapeutic agents highlights their potential as novel diagnostic biomarkers and next-generation therapeutics in cardiology [223]. Hu et al. demonstrated that Treg-derived exosomes significantly ameliorate acute myocardial infarction (AMI) by reducing infarct size, improving cardiac function, and suppressing cardiomyocyte apoptosis. In the mouse model used, inhibition of exosome release (using GW4869) abolished the cardioprotective effects of Tregs, whereas administration of purified Treg-derived exosomes replicated these benefits, including decreased myocardial injury markers and enhanced left ventricular performance. Mechanistically, exosomes derived from Treg suppressed the expression of M1 macrophage markers and promoted polariztion to M2 macrophage markers in myocardial tissues of AMI mice. Importantly, macrophage depletion eliminated these therapeutic effects, confirming that exosome-mediated immunomodulation of macrophages is central to the cardioprotective mechanism. Overall, this study demonstrates that Treg-derived exosomes may serve as a promising treatment strategy for ischemic heart diseases and AMI [146].

## 5 Current challenges and future directions

Although CAR-T cell therapy has demonstrated significant efficacy in clinical trials targeting relapsed or refractory CD19-positive B-cell malignancies, these treatments are accompanied by specific toxicities that may be severe or even fatal [224]. Multiple clinical studies have reported adverse events, such as cytokine release syndrome, infections, febrile neutropenia, and neurological toxicities [[225], [226], [227], [228]]. Cytokine release syndrome and neurotoxicity are recognized as the two most prevalent toxicities associated with CAR-T cell therapies, particularly those targeting CD19 [229]. In contrast, the manifestation of on-target, off-tumor effects, such as B-cell aplasia in hematological malignancies, is generally manageable and does not pose a lethal risk to patients. However, off-tumor toxicities significantly impact the application of CAR-T cell therapies in the context of solid tumors. Enhancing safety and regulating the supraphysiological activity of CAR-T cells is of paramount importance. Innovative control strategies has been developed to improve the safety outcomes. These strategies include switch-based control systems, microenvirinmental stimuli responsive CARs, and combinatorial antigen recognition methods [230]. In switch-based control systems, the external switch molecules modulate the activity of CAR-T cells, which can either suppress T cell activity via elimination switches or enhance T-cell cytotoxicity via the implementation of ON-switch CAR systems or bi-functional intermediate switches. Combinatorial strategies using AND- and NOT-gate circuits have been developed to mitigate the risk of on-target, off-tumor toxicities. These circuits enhance the specificity of T cells to distinguish between target and bystander cells. In NOT-gate circuits, CAR-T cells can bypass self-activation when bind to non-cancerous cells. In contrast, AND-gate dual-receptor systems promote CAR-T cells activation rather than inhibition by providing complementary signals [230,231]. An alternative approach to enhancing safety involves CARs that are activated exclusively upon encountering specific physical characteristics within the tumor microenvironment. This strategy enables tumor site-specific activation of CAR T cells. Masked CARs is a notable example of this strategy, which incorporate a masking peptide linked via a protease-cleavable linker that conceals the extracellular domain of the CAR [232]. Additionally, another microenvironmental feature exploited for localized CAR-T cell activation is the hypoxic condition caused by the aberrant vasculature of tumors. These specialized CARs contain an oxygen-sensitive subdomain that remains stable at low oxygen level but undergoes degradation in normoxic environments [233].

To date, CAR-T cells used in clinical trials have predominantly been derived from autologous T cells. The favorable safety profile of CAR-T cells and T cell-based drug delivery systems stems from this patient-specific approach, which minimizes immunological risks such as rejection and graft-versus-host disease. However, autologous T cells have inherent limitations because the therapeutic product must be generated from each individual patient's cells. This process is time-consuming, costly, and carries a risk of manufacturing failure. Such delays in treatment availability are especially critical for patients with rapidly progressing diseases. Additionally, the quantity and quality of the initial autologous T cells present further challenges, as patients often undergo lymphodepleting chemotherapy and/or radiotherapy prior to cell collection [234]. To enable successful clinical implementation, it is essential to employ advanced technologies that ensure consistent product quality across multiple geographically distributed manufacturing sites. This strategy includes integrating automated cell production systems, real-time sensor monitoring, and process simulation techniques to improve quality control and optimize supply chain efficiency [235]. Furthermore, the need for lymphodepleting chemotherapy and its associated adverse effects can be overcome with help of in vivo T cell engineering. This strategy involves the direct administration of nucleic acids to circulating T cells of patients with viral or non-viral vectors. The ribosomes then translate the delivered nucleic acid to express CARs or TCR on T cell surface. This technique may also promote epitope spreading by preserving an intact immune system, thereby enhance a robust anti-tumor response [89].

The clinical application of T cell hitchhiking faces various challenges in obtaining regulatory approval. For effective and safe implementation of this approach in clinical settings, a series of standardized and comprehensive evaluations must be performed beforehand. These evaluations should include: (i) Immunogenicity assessment: It is essential to thoroughly evaluate the potential immune responses elicited by the hitchhiking system, particularly when nanoparticles or foreign substances are involved [164]. (ii) Biocompatibility studies: These studies will identify the toxicity to cells, tissus, and possible immune response, which will help in refining the system for safety and efficacy [37]. (iii) In vivo studies: in vivo investigation within living organisms gurantee the clearence and distribution of nanomaterials hitchhiked to the circulating cells. Careful execution of these tests will help in resolving regulatory concerns that is required for successful translation of hitchhiking system [37]. (iv) Long-term safety and toxicity: Researchers must investigate the degradation of hitchhiked nanomaterials over time for potential adverse effects [164]. Future research should focus on targeting spcificity, controlled drug release, and scalability and manufucturing [130,236].

Regarding T cell-derived exosomes, several hurdles remains to be overcome before their transition from bench to bedside. First, the methods of isolating and characterizing exosomes vary between laboratories. Therefore, standardized protocols for the isolation, purification, and therapeutic use of exosomes need to be developed. The second challenge is the production of exosome, which are very small and it is often difficult to produce in quantities sufficient for drug delivery applications. Consequently, large-scale production methods are required to meet the demands of drug delivery. Furthermore, the toxicity and stability of exosomes after drug loading or modification need to be further investigated, especially when used as vectors for tumor nanomedicines. Addressing these issues will facilitate the clinical translation of exosomes-based therapies [237].

T cell membrane-coated nanoparticles demonstrate significant potential for addressing various disease conditions; however, several challenges associated with this technology must be overcome. A critical aspect of this approach is ensuring that membrane proteins retain their functionality, which requires the proper orientation and complete coating of nanoparticles with cell membranes. Research indicates that nearly 90% of the core nanoparticles are only partially enveloped by the plasma membrane [238]. Establishing the biocompatibility of T cell membrane-coated nanoparticles is required for advancing to clinical trials. Although short-term biocompatability has been proved by various preclinical investigations, significant challenges must be addressed before these nanomaterials can be utilized in clinical applications. The accumulation of these substances in healthy tissues presents potential risks, and engineering T cell membranes may heighten health concerns by triggering hyperinflammatory responses through the release of inflammatory mediators [13]. Therefore, a more comprehensive understanding of the interactions between cellular membrane components and the biological environment is essential. Furthermore, the mechanisms governing the release of cargo from nanoparticles camouflaged with T cell membranes remain poorly understood. Elucidating the drug release mechanisms is essential for advancing the development of these nanocarriers. Bridging experimental investigations with molecular simulations, a comprehensive understanding of the endocytic entry mechanisms and drug release processes for these nanoplatforms can be achieved [239]. Another critical issue that must be addressed prior to clinical translation of T cell membrane-coated nanoparticles is the larg-scale production of this technology. The current lab-based production techniques are extrusion, sonition, and microfluidic. Each one of these technique has pro and con. For example, the extrusion technique leads to high uniformity but low yield efficiency. Therefore a straightforward, relaible and standardized protocol is required for large-scale production this technology [40]. Furthermore, the variability in characterization and purification protocols for plasma membranes across different laboratories leads to inconsistencies in the physico-chemical properties of T cell membranes. Therefore, a consistent and highly reproducible techniques are crucial for evaluating cell membrane integrity.

## 6 Conclusions

In conclusion, T cell-mediated therapeutic delivery strategies—such as T cell membrane-coated nanoparticles, T cell-derived exosomes, T cell hitchhiking, and CAR T cells—represent innovative targeted platforms with broad applications across various pathological conditions. These approaches leverage the intrinsic targeting, immune modulation, and homing capabilities of T cells to enhance delivery specificity, reduce systemic toxicity, and improve therapeutic efficacy. However, several limitations currently hinder their widespread clinical application. Looking ahead, future research should prioritize enhancing the robustness and scalability of techniques for isolating and engineering cell membranes and exosomes to ensure consistent quality and safety. Advances in bioengineering, such as glycoengineering and ligand modification, could improve targeting specificity and the delivery of therapeutic payloads. The development of less invasive in vivo cell modification techniques may mitigate immune rejection and preserve the functionality of carrier cells in hitchhiking strategies. For CAR-T cells, innovations aimed at reducing toxicities, overcoming tumor resistance mechanisms, and expanding indications beyond hematologic malignancies are essential. Furthermore, integrating combinatorial therapies and personalized medicine approaches may optimize therapeutic outcomes. Overall, sustained interdisciplinary research and clinical assessment are crucial for overcoming current limitations and fully harnessing the potential of T cell-mediated drug delivery for precision medicine across a range of disease states.

## Declaration of generative AI and AI-assisted technologies in the writing process

During writing this review article, the author(s) used WORDVICE.AI in order to improve English language. After using this tool/service, the author(s) reviewed and edited the content as needed and take(s) full responsibility for the content of the published article.

## CRediT authorship contribution statement

**Nasrullah Jan:** Conceptualization, Project administration, Software, Supervision, Writing – original draft, Writing – review & editing. **Hassan Shah:** Writing – original draft. **Safiullah Khan:** Writing – original draft. **Naveed Ullah Khan:** Data curation, Software. **Yan Li:** Writing – original draft. **Xinwei Zhang:** Formal analysis, Funding acquisition. **Guixiu Shi:** Supervision, Writing – review & editing.

## Declaration of competing interest

The authors have no any conflict of interest.

## Data Availability

No data was used for the research described in the article.
